# The take-off of the Saudi professional football league in the context of the 2030 vision: effect on the competitive balance

**DOI:** 10.3389/fspor.2025.1649310

**Published:** 2025-09-26

**Authors:** Mohammed Jamal Bataineh, Juan Carlos Guevara-Pérez, Emilio Martín-Vallespín, Rudemarlyn Urdaneta-Camacho

**Affiliations:** ^1^ Faculty of Business Studies, Arab Open University, Riyadh, Saudi Arabia; ^2^ Department of Accounting and Finance, Faculty of Economics and Business, University of Zaragoza, Zaragoza, Spain

**Keywords:** football, Saudi arabia "Vision 2030", resource dependency theory, competitive balance, market competition

## Abstract

**Introduction:**

This study examines the impact of Saudi Arabia's Vision 2030 on the competitive balance of the Saudi Professional League (SPL), focusing on how the entry of public investment funds (PIF) into club ownership has transformed the league's structure.

**Methods:**

Competitive balance is measured using the Herfindahl Index of Competitive Balance (HICB), which captures the degree of concentration in the distribution of league points across ten seasons (2014/15–2023/24).

**Results:**

Results indicate that the SPL's growth has been accompanied by greater imbalance between clubs, reducing competitive uncertainty and, potentially, spectator appeal. The main issue is not the inflow of capital or foreign players per se, but their concentration in a small number of teams.

**Discussion:**

Despite the deterioration in competitive balance, driven by widening financial disparities between a small group of clubs and the rest, the SPL has significantly increased its international visibility. This pattern appears consistent with the priorities of Vision 2030 to enhance the national brand image and soft power, although it may delay progress towards financial sustainability at the league level. At the same time, football can amplify the visibility and agency of local communities by creating spaces for participation and inclusion, suggesting that targeted grassroots initiatives could help translate international profile into durable domestic support. While debates about “sportswashing” persist, the broader policy intent of Vision 2030 aligns with the UN's 2030 Agenda emphasis on diversification. Ultimately, any durable impact should be assessed against measurable future social and economic outcomes.

## 1 Introduction

Over the last five years, the Saudi Professional Football League (SPL) has undergone an unprecedented transformation, marked by high-profile transfers of internationally renowned players such as Cristiano Ronaldo, Benzema, Neymar and Mané, among others. This sudden interest in football in Saudi Arabia replicates, to some extent, the steps previously taken by Qatar. Similar to its neighbouring country, Saudi Arabia is combining foreign investments—such as the acquisition of historic European clubs like Newcastle United—with ambitions to host major global sporting events, including the 2034 FIFA World Cup. At the heart of this strategy lies a significant domestic effort to elevate the SPL into one of the ten most valuable football leagues in the world within the short term.

According to Transfermarket data^1^ between the 2021–22 and 2023–24 seasons, the Saudi Professional League increased its estimated market value from approximately €370 million to €970 million. During this period, clubs such as Al-Ahli SFC, Al-Hilal SFC, and Al-Nassr FC—home to Cristiano Ronaldo—saw their market values rise by approximately 450%, 330%, and 250% respectively. Together, these three clubs accounted for a combined estimated market value of €499 million. This expansion has been driven primarily by the Saudi Arabian Public Investment Fund (PIF), which holds a 75% stake in the country's four largest clubs.

This strong state involvement raises critical questions about the underlying objectives of such investment. Is the primary goal to transform the SPL into a globally competitive league, or is it part of a broader geopolitical strategy of “sportswashing” using high-profile sports events and investments to improve the country's international image? Saudi Arabia's broader strategy, articulated in its Vision 2030 plan, also includes ventures like LIV Golf and the hosting of international events such as the Spanish Super Cup, further blurring the line between economic development and political soft power.

This study does not aim to adjudicate these debates. As others have argued, sportswashing is a diffuse, polysemic, and selective concept often more useful for activism and media rhetoric than for cumulative academic theory (1). While it highlights states' efforts to improve their reputation through sport, it does not provide a coherent new analytical lens in relation to previous notions such as propaganda, public diplomacy or place branding (2).

The rapid, top-down expansion of the SPL, largely supported by public capital, brings into focus longstanding debates in the economics of sport. These include concerns over the efficiency and accountability of public funding in achieving its objectives, as well as the potential for such investments to disrupt competitive equilibrium within the league. When certain clubs benefit from disproportionate financial backing, questions inevitably arise about fairness, sustainability, and the integrity of competition.

Accordingly, this paper examines the effects of public investment in the SPL with particular attention to competitive balance as a core indicator of league health and attractiveness. We also situate the analysis within the broader context of domestic engagement and governance under Vision 2030.

The rationale of the study is to assess how Saudi Arabia's Vision 2030 (implemented through state-led, PIF-backed investment in the SPL) has boosted market value and international visibility while raising concerns about resource concentration and long-term sustainability. To address the limited empirical evidence on these dynamics and their intersection with ongoing social and political change, we track competitive balance using the NHICB across ten seasons (2014/15–2023/24) and interpret the results within Vision 2030's governance and community-engagement objectives, in order to evaluate the sustainability of the expansion path and its implications for broader transformation in the Kingdom.

## 2 Context and theoretical framework

### 2.1 Football as an environmental, social, and governance (ESG) strategic factor within the framework of Saudi Arabia's “Vision 2030”

Since 2014, oil-dependent countries in the Middle East and North Africa have faced significant challenges due to a sharp decline in oil prices. Despite warnings from organizations such as the International Monetary Fund (IMF) urging these nations to diversify their sources of revenue, many Gulf countries have struggled to transition away from oil dependency. This difficulty stems from deeply entrenched interests that benefit from oil revenues and the presence of weak state institutions (3). In response to these challenges, Saudi Arabia launched its Vision 2030 initiative in 2016, under the leadership of Crown Prince Mohammed bin Salman. This strategic framework aims to reduce the Kingdom's reliance on oil, diversify the economy, and modernize public administration.

Social changes in Saudi Arabia, such as those promoted under Saudi Vision 2030, are often initiated from the top down through formal decrees that establish new behavioral norms (4). Landmark reforms, such as allowing women to drive in 2017 or to travel without male guardian permission after the age of 21 in 2019, were initially perceived as paradigm shifts that signaled a broader redefinition of everyday social boundaries (5). In this sense, these authors point out that the implementation of these transformations has not been without social tensions, as traditional values sometimes clash with Western-influenced models of social norms that emphasise individualism and global inclusion.

Vision 2030 is structured around three main pillars: a vibrant society, a thriving economy, and an ambitious nation (6). Among its goals are increasing non-oil government revenues, enhancing the role of the private sector, and improving quality of life through health, education, and entertainment reforms. The Saudi Vision 2030 strategy aligns with a broader regional trend in which Gulf Cooperation Council states are deploying sport and cultural initiatives to bolster soft power and diversify beyond oil-dependent economies. As AlSaeed (7) observes, countries such as Saudi Arabia, Qatar (Qatar National Vision 2030), and the UAE (UAE Vision 2021) deploy sports diplomacy—particularly football—as a strategic tool to enhance their international standing and stimulate economic engagement This is visible in Qatar's hosting of the FIFA World Cup 2022 and acquisition of football clubs like Paris Saint-Germain, as well as the UAE's investment in the City Football Group and hosting of events such as the Abu Dhabi Grand Prix. Bahrain, through its involvement in Formula One, and Oman, with its Oman Vision 2040, are also pursuing similar strategies, albeit on a smaller scale. These initiatives are intended to reinforce the nation branding of these countries by promoting a more modern image, strengthening international confidence, and enhancing both national pride and global reputation (8, 9).

Sport, and particularly football, is framed as a core mechanism to promote healthier lifestyles, increase civic engagement, and elevate Saudi Arabia's global image (9, 10). To operationalize Vision 2030, the government implemented the National Transformation Program (NTP) and the Fiscal Balance Program (FBP). These aim to reduce fiscal dependency on oil and stimulate non-oil sector development. As part of this agenda, sport is identified as a high-impact sector for public investment and image diplomacy. In this context, football has emerged as a flagship project for both domestic reform and global visibility. According to the Vision 2030 Annual Report (6), early phases of implementation have already yielded measurable outcomes in areas such as private sector participation, regulatory reform, and the diversification of entertainment and sports offerings, including the expansion of the Saudi Professional League.

The institutional foundation of football in Saudi Arabia has been shaped by the Saudi Football Federation (SFF), established in 1956. The SFF governs over 150 clubs and oversees national teams at all levels. Over the years, Saudi football has made notable strides, including multiple Asian Cup victories and consistent qualifications for FIFA World Cups. The federation has also introduced reforms aligned with Vision 2030, such as adopting a new emblem and embracing digital strategies to enhance fan engagement and generate commercial value (11, 12). In line with these ambitions, Saudi Arabia is set to host the FIFA World Cup in 2034, a strategic milestone through which the Kingdom aims to replicate the soft power successes of South Korea in 2002 and Qatar in 2022 (9). This global event is expected to further elevate Saudi Arabia's international profile and position the country as an emerging leader in the global football ecosystem, reinforcing the broader objectives of Vision 2030.

Indeed, Qatar's 2022 experience constitutes a relevant precedent for understanding the potential of mega sporting events in Arab contexts, as the tournament managed to consolidate benefits in terms of international exposure, strengthening internal social cohesion, and gaining diplomatic legitimacy through its demonstrated organizational capacity (13–15). These findings suggest that the outcomes observed in Qatar's 2022 could be replicated in Saudi Arabia's bid for 2034, illustrating not only the opportunities to strengthen regional identity, project cultural and economic leadership, diversify its international image, and attract foreign investment, but also the necessary conditions to proactively manage criticism and maximize social and cultural legacies.

In the case of Korea/Japan 2002, the tournament's positive effects have improved South Korea's international image as a tourist destination (73). Additionally, the momentum generated by the 2002 FIFA World Cup has contributed to the difficult process of reconciliation between the two countries since World War II, contributing to their reconciliation (74).

One of the most symbolic actions under Vision 2030 was the acquisition of majority stakes in Saudi football clubs by the PIF, which manages assets exceeding $800 billion. The PIF has driven high-profile signings that have rapidly increased the international visibility of the Saudi Professional League (SPL). A key turning point was the signing of Cristiano Ronaldo by Al-Nassr FC in late 2022. His arrival triggered a sharp rise in global interest: the club's Instagram following surged by over 400%, SPL match attendance increased, and the league signed broadcasting deals expanding its reach to over 30 new countries (16). This illustrates how football under Vision 2030 is not only being used as a policy tool for health and leisure but also as a powerful instrument of global influence and soft power.

In parallel, the Ministry of Sport has also approved the privatization of football clubs as part of the drive to transition away from government control and towards financial independence. This reform aims to bring Saudi clubs in line with global best practices and to professionalize governance, talent development, and financial sustainability (17). The goal is to eventually attract private investors and reduce reliance on public funding.

This strategy aligns, in part, with the United Nations' Agenda 2030, particularly in its focus on sustainability, economic diversification, and improving quality of life through sport. While the UN Agenda 2030 pursues these goals from a global, inclusive perspective, Saudi Arabia adapts similar principles within a national framework that combines economic modernisation and international branding (18). Both initiatives recognize sport as a driver of social development, international cooperation, and economic opportunity (9, 19).

Nonetheless, critiques of Saudi Arabia's sport strategy have emerged. Some scholars view these investments as part of a broader sportswashing agenda—leveraging sport to reshape global perceptions without addressing deeper structural issues such as governance or human rights (20, 21). At the same time, the term remains analytically ambiguous and is often applied selectively to non-Western or emerging hosts (e.g., Russia, India, Saudi Arabia, China). However, a more consistent application would also encompass events in advanced democracies where social or human-rights controversies persist (1). London 2012, for instance, was read abroad as image-building and at home as a distraction from unpopular austerity measures (22, 23). For these reasons, sportswashing is not a central theme of this study; we invoke it only to indicate how sport can simultaneously legitimise and expose political projects, which is the relevant context for interpreting the SPL within the framework of Vision 2030 (2, 24).

In any case, it is important to make clear the speculative nature of these types of statements, which, being more prejudiced than predictive, still require a maturing horizon to transcend activism and media rhetoric to provide empirical evidence within academic theory (1). The long-term impact of these policies will therefore depend not only on financial investment and international success but also on genuine structural reform and sustainable growth across all levels of football.

### 2.2 Football as a strategy of Saudi Arabia's “Vision 2030” within the framework of the resource dependency theory

The expansion of the Saudi Professional League (SPL) within the framework of Vision 2030 can be more deeply understood through the lens of Resource Dependency Theory (RDT). The RDT provides a compelling lens through which to analyze both the opportunities and constraints of the SPL's transformation. Originally formulated by Pfeffer and Salancik (25), RDT posits that the survival and effectiveness of organizations depend largely on their ability to acquire and manage critical external resources. These dependencies shape organizational behavior, strategic alliances, and governance models.

In this context, football clubs—particularly in emerging markets like Saudi Arabia—do not operate in isolation. Their capacity to attract talent, develop infrastructure, and compete on a global stage is heavily influenced by their access to financial, political, and social resources. In contrast to more mature football economies, such as those in England, Spain, or Germany, where clubs benefit from diversified revenue streams (e.g., broadcasting rights, commercial deals, and fan engagement), many clubs in the Saudi Pro League (SPL) remain structurally dependent on state funding. This public resource flow is primarily managed through the PIF, which holds a 75% stake in the country's four most prominent clubs (Al-Hilal SFC, Al-Nasser FC, Al-Ahli SFC and Al-Ittihad Club). Such a level of financial centralization creates a clear pattern of organizational dependency (17). According to RDT, this dependence does not merely determine the survival of clubs but also affects their strategic direction and governance autonomy (26). In the Saudi case, the state—via the PIF—becomes the central actor in shaping the evolution of professional football. This allows the government to align club development with broader policy objectives under Vision 2030, such as enhancing the country's international image, attracting tourism, and promoting social engagement through sport.

However, this form of centralized funding also presents structural risks. As observed in the academic literature, dependency on a dominant external benefactor—sometimes referred to in sports economics as a “sugar daddy” model—can create short-term success at the expense of long-term sustainability (27, 28). When resources are allocated without regard to market performance or financial self-sufficiency, clubs may become vulnerable to political shifts, changing state priorities, or economic downturns. Similar patterns have been observed in Europe under UEFA's Financial Fair Play regulation, where dependency on wealthy benefactors has led to instability once external funding was reduced or withdrawn (29, 30).

Furthermore, RDT helps explain why Saudi Arabia's investment in football is not limited to club financing, but extends to governance control and institutional influence. The board composition of Saudi clubs remains heavily influenced by government-appointed executives, limiting the development of autonomous, commercially driven management structures (31, 32). This tight linkage between state and sport reflects what RDT describes as an exchange relationship: clubs receive essential resources but, in return, contribute to national goals, such as the image repositioning of Saudi Arabia under Vision 2030.

As the SPL moves toward privatization, one of the cornerstones of Saudi Arabia's football reform, the key challenge will be to reduce this dependency and foster more diversified and sustainable business models. This transition—from a rentier model to a competitive market-driven system—requires clubs to develop internal capabilities for revenue generation, such as marketing, fan engagement, and international partnerships (17, 33). Privatization, in this context, is not merely an economic reform but a structural realignment intended to rebalance power dynamics and reduce institutional vulnerability.

Moreover, RDT offers a valuable framework for evaluating the broader implications of Saudi football's financial architecture on competitive balance. When only a select group of clubs benefit from concentrated public investment, inequalities across the league deepen, making competition less predictable and potentially reducing fan engagement over time. In contrast, if resource flows are conditioned by performance and efficiency, RDT would predict a healthier organizational ecosystem with more equitable competition (34, 35).

While RDT provides a useful lens to explain the SPL's reliance on state funding and centralized governance, its applicability is not universal. As Hillman et al. (75) state, “research has been slower to determine the boundary conditions of RDT—that is, when it is more or less predictive.” The theory works best in situations of strong, unavoidable external dependency, but becomes less relevant when organizations can access alternative resources or operate with greater autonomy. In the SPL, current dependence on the PIF clearly fits RDT's assumptions. However, if privatization and revenue diversification through broadcasting rights, merchandising, and sponsorships reduce this dependency, other frameworks—such as institutional theory or stakeholder theory—may better explain future developments. Recognizing these boundary conditions helps anticipate how changes in the SPL's resource environment will shape its sustainability under Vision 2030.

The current state-led model has enabled rapid growth and international projection of Saudi football. However, unless accompanied by reforms that diversify resource acquisition and strengthen internal management structures, the long-term sustainability of SPL may be compromised. A successful shift away from dependency will be essential for ensuring that sport fulfills its intended role within Vision 2030—as a sustainable pillar of economic diversification, social development, and global influence.

### 2.3 Impact of the “Vision 2030” project on the competitive balance and market competition of the Saudi professional league

An essential feature of professional football's appeal lies in competitive uncertainty—the possibility that any team can win on any given day. This principle underpins competitive balance, a concept essential for maintaining the attractiveness, fairness, and commercial viability of a league (36). When match outcomes become predictable due to deep structural inequalities, leagues risk losing fan interest, commercial investment, and sporting integrity (37).

The Saudi Professional League (SPL), within the framework of Vision 2030, faces a complex challenge: sustaining competitive balance while accelerating growth through state-led investment. The current strategy has enhanced international visibility, but the concentration of resources in a small number of clubs (specifically Al-Hilal SFC, Al-Nassr FC, Al-Ahli SFC, and Al-Ittihad Club) mainly through PIF financing, has increased the disparity between top-tier and lower-tier teams. This concern is echoed in academic literature, which has identified several interrelated factors that shape competitive balance across domestic football leagues. The following points present these factors and provide a framework for assessing how Vision 2030 reforms and PIF-led investments may shape competition in the Saudi professional football market (see Table 1):

a) League Regulations and Governance Structures

**Table 1:** Summary of competitive balance factors in the Saudi professional league (SPL).

Factor group	Key aspects and Saudi context
1. League Regulations and Governance	SPL lacks unified redistributive policies; promotion/relegation system exists, but regulation is nationally controlled without supranational harmonization.
2. Financial and Economic Factors	Heavy PIF investment benefits a few clubs; absence of revenue sharing and reliance on state funds intensifies financial inequality.
3. Talent Distribution and Player Market	High-profile players like Ronaldo concentrated in elite clubs; talent is not evenly distributed due to unequal financial power.
4. Performance and International Competition	Top clubs gain prestige and income through AFC participation; growing disparity in media attention and drawing power.
5. Financial Regulation (FFP-like mechanisms)	No formal FFP, but unchecked investment risks long-term imbalance; potential need for future regulation to ensure sustainability.

Source: Author elaboration.

League regulations play a central role in shaping the distribution of competitiveness. National leagues often adopt regulatory frameworks tailored to domestic political and economic preferences, including how revenues are shared and how competition is structured (35). Attempts to harmonize such rules across borders, such as within UEFA through financial fair play (FFP) rules, may face resistance for overriding national interests.

Institutions like UEFA, the Asian Football Confederation (AFC) or the CONMEBOL are essential in resolving coordination failures (e.g., prisoner's dilemma scenarios) that emerge when leagues act independently, potentially weakening overall balance (38). Additionally, internal rules such as the number of participating teams, the existence of promotion/relegation systems (open vs. closed leagues), and the competition format (e.g., playoffs vs. round-robin) can significantly influence balance. While open leagues with promotion and relegation are common in Europe and the Middle East, empirical results regarding their effect on balance are mixed (39).

b) Financial and Economic Factors

Economic disparities between clubs are one of the most critical determinants of imbalance. In theory, revenue sharing mechanisms—whether from broadcasting rights, commercial sponsorships, or ticket sales—can help mitigate disparities. Leagues like the National Football League (NFL) in the United States and Germany's Bundesliga have adopted relatively egalitarian models that promote parity (40–42), while others like the Premier League face ongoing concentration of income. In the SPL, no significant income redistribution mechanisms exist, and public funding has largely been discretionary. As a result, differences in wage budgets, infrastructure, and market power persist. For example, league data reports that Al-Hilal SFC's total expenses reached SAR 640 million in the 2022/23 season, while Damac FC's expenses were SAR 85.9 million,a difference of more than sevenfold (43). Moreover, macroeconomic factors such as national GDP influence league strength. A higher GDP correlates with increased capacity to retain top domestic talent and attract foreign stars, enhancing competitive parity at the international level (42, 44).

Taxation policies, financial oversight, and regulations on external investments also matter. In Saudi Arabia, the dominant role of the PIF has shielded clubs from market forces, reinforcing dependency and unequal growth. Finally, international prize money from competitions such as the AFC Champions League may exacerbate inequality by rewarding already dominant teams with additional resources.

c) Talent Distribution and Player Market Dynamics

The distribution of talent is a key factor influencing balance (45). Studies show that access to foreign players can improve the quality and parity of a league, especially when lower-tier teams gain access to skilled imports (21, 41). The SPL has witnessed a significant inflow of foreign talent; while all clubs employ foreign players, high-profile players from top European sides are largely concentrated in clubs receiving substantial public investment. The arrival of Cristiano Ronaldo at Al-Nassr is an illustrative example. While his presence boosted the club's global profile and helped attract attention to the league, it also magnified existing inequalities. Clubs without comparable financial backing have little chance of acquiring equivalent players, creating a clear segmentation in competition (16). Other influential factors include the mobility of players, salary caps, draft systems, and the depth of the talent pool. While mechanisms like drafts and caps have been effective in North American leagues, they are largely absent in Saudi football (46).

d) Performance Incentives and International Competition

Participation in international tournaments introduces new dynamics (40, 47). While it boosts revenue and reputation, it may also widen the gap between domestic champions and mid- or lower-table teams. Rocaboy (48) argues that the trade-off between domestic balance and international competitiveness is a structural tension in modern football. Additionally, “drawing power"—the capacity of clubs to attract fans, sponsors, and media—can distort league equilibrium. When a few clubs monopolize attention and revenue, competitive balance suffers (49). This trend is seen in many European leagues and is emerging in the SPL, where Al-Hilal, Al-Nassr, and Al-Ittihad dominate both commercially and on the pitch.

Interestingly, perceived competitive balance is sometimes more important than actual balance. Fans may remain engaged if multiple teams compete for titles or survival, even if the league is unbalanced overall (36). However, sustained dominance by a single club—or even a duopoly—can erode long-term fan interest and reduce the league's appeal.

e) Financial Fair Play (FFP) and Regulatory Models

In Europe, UEFA's Financial Fair Play (FFP) regulations were introduced to enhance financial stability. However, several studies have found that FFP may inadvertently entrench existing inequalities, acting as a relative salary cap that limits upward mobility (35, 37). By restricting deficit financing, smaller clubs are less able to invest in growth, while larger clubs with established revenues face few constraints (50).

Although the SPL does not currently operate under a formal FFP regime, any future implementation of financial control mechanisms must balance stability with inclusivity. Without effective oversight, current investment patterns risk institutionalizing inequality and reducing the SPL's long-term competitiveness.

## 3 Methodology

The methodology for measuring competitive balance is based on the Herfindahl Index of Competitive Balance (HICB) designed by (51) which measures the degree of concentration in the distribution of total points scored in a league. The Herfindahl-Hirschman Index (HHI) is widely recognized as a valid and robust indicator of competitive balance in football leagues. Its simplicity, transparency, and comparability across different leagues and competitions have led to its frequent application in sports economics research, including studies by Ramchandani et al. (50) on Europe's top football leagues and Ávila-Cano et al. (52) on the Six Nations Rugby Championship. For each season, the SPL HICB scores are calculated using the following formula:

HICB=(HHI/(1/n))100

Where HHI is the sum of the squares of the points quota of each club playing in a league in each season and n is the number of teams in that league and season. The case of maximum equilibrium would be that season in which all teams score the same number of points, and the index would take a value of 100. As the index increases, the competitive equilibrium decreases. Table 2 shows a practical example of the HICB calculation for the 2022/23 season. As can be seen, Al Ittihad FC scored 72 points, representing 10.7% (0.107) of the total points scored by the 16 SPL teams (670 points). The sum of the square of the percentage of points obtained by each team represents the HHI and when multiplied by 16, the HICB is obtained, which in this case gives a value of 112.23.

**Table 2:** HICB of the 22/23 season.

Post	Club	Pts	% points	% points squared
1	Al Ittihad FC Jeddah	72	0,107	0,012
2	Al Nassr FC	67	0,1	0,01
3	Al Hilal FC Riyadh	59	0,088	0,008
4	Al Shabab FC Riyadh	56	0,084	0,007
5	Al Taawon FC	55	0,082	0,007
6	Al Fateh SC	43	0,064	0,004
7	Al Ittifaq FC Dammam	37	0,055	0,003
8	Dhamk Club	36	0,054	0,003
9	Al Raed Club	34	0,051	0,003
10	Al Tai Ha'il	34	0,051	0,003
11	Al Feiha	33	0,049	0,002
12	Abha Club	33	0,049	0,002
13	Al Wahda FC Mecca	32	0,048	0,002
14	Al Khaleej Saihat	31	0,046	0,002
15	Al Adalh Club	28	0,042	0,002
16	Al Batin Club	20	0,03	9E-04
	TOTAL POINTS	670	1	**112,23**

Source: Own's elaboration.

Despite its strengths, the Herfindahl Index of Competitive Balance (HICB) has several limitations. As it is calculated season by season, it does not capture the evolution of club dominance over time, that is, whether top-performing teams rotate or the same clubs consistently dominate (53). Another drawback is that the HICB ignores competitive proximity, meaning how closely clubs are grouped in different parts of the standings. In this regard, fans tend to perceive competitiveness through “sub-competitions” (such as the title race, relegation battle, or qualification for continental tournaments), rather than from overall balance alone (38, 51).

The sample is composed of the teams participating in SPL football over the last decade, seasons 2014/15 to 2023/24. During this period the SPL has experienced a remarkable increase in the market value of its clubs, highlighted by the gradual arrival of some international players.

Another limitation of the HICB is its sensitivity to the number of participating teams, which can hinder comparisons across seasons in which the league changes its size, thereby requiring adjustments to normalize the results. In the case of the SPL, as the size of the competition has been extended twice, from 14 to 16 in the 18/19 season and from 16 to 18 in the 23/24 season, it is necessary to apply a normalisation factor to the HCIB. This approach follows the method used by Ramchandani et al. (50) to facilitate the comparison between seasons with different league sizes. For this purpose, the HCIB becomes the Normalised Herfindahl Index of Competitive Balance (NHICB) using the following formula:

NHICB=HICB×MaxHICB(16)/MaxHICB(n)

Where n is the number of teams in the SPL in a given season, Max HICB(n) is the upper threshold of HCIB that would be reached by the most unbalanced distribution of points in a season with n clubs, and Max HICB(16) is the maximum HICB that can be reached with the most unbalanced distribution of points in a season with 16 clubs. The value of Max HICB(16) is 137.77 while Max HICB(14) is 138.48 and Max HICB(18) is 137.25. Thus, to normalise the results to those of a 16-team season, the HICB of 14-team leagues will be corrected by the coefficient (137.77/138.48) and the HICB of the 2023–24 season with 18 teams will be adjusted using the coefficient (137.77/137.25).

In addition to the Herfindahl Index of Competitive Balance (HICB), the study carries out a comparative analysis of financial disparities by calculating the ratio between the total market value^2^ of the four most valuable clubs and the four least valuable clubs in the Saudi Professional League (SPL). This approach allows for a clearer understanding of the concentration of economic power within the league and its implications for competitive balance. Existing literature supports the use of market value as a valid proxy for a club's financial capacity and sporting potential, given its close correlation with transfer spending, wage bills, and on-field performance (27, 35). Moreover, several studies have adopted similar ratio-based methods to measure inequality and its effect on league dynamics (38, 54). By comparing extreme tiers of the league, this measure complements traditional indices and provides a more intuitive, economically grounded assessment of structural imbalance in the SPL.

Finally, a correlation analysis was conducted using the variables presented in Table 3 in order to explore potential statistical relationships between competitive balance (as measured by the NHICB), the overall market value of the league, and the Q1/Q4 ratio, which reflects the disparity between the most and least valuable clubs**.** Given the small sample size and the potential non-normality of the data, Spearman's correlation coefficient was considered more appropriate for assessing the relationships between variables.

**Table 3:** HICB developments.

Season	HICB	NHICB	Market value (millions €)	Q1/Q4
2014/15	116.4	115.8	119.22	9.06
2015/16	114.8	114.2	105.08	4.05
2016/17	115.0	114.4	169.06	5.17
2017/18	108.5	108.0	207.39	3.15
2018/19	112.2	112.2	424.46	6.14
2019/20	109.0	109.0	376.7	5.47
2020/21	107.9	107.9	384.1	5.69
2021/22	110.8	110.8	370.4	5.52
2022/23	112.2	112.2	349.93	6.05
2023/24	114.87	115.3	993.45	13.33
Average	112.2	111.98	278.5	5.6

Source: Author's elaboration.

## 4 Results

The results of the study show an average NHICB of 111.98 across the ten seasons analyzed. Overall, two distinct periods with different trends can be identified: the first from the 2014/15 to the 2017/18 seasons, and the second beginning in 2018/19. The first period, when the SPL had 14 teams, is characterized by a significant decline in the NHICB, indicating an improvement in competitive balance. During these four seasons, the league nearly doubled its market value while financial disparities among clubs narrowed. This trend is further illustrated by the reduction in the disparity ratio between the most and least valuable quartiles of clubs (Q1/Q4), which dropped from 9.06 in 2014/15 to 3.15 in 2017/18 (see Table 3). This phase reflects a period of organic growth, where investments were more evenly distributed and clubs were less dependent on external resources—consistent with the principles of Resource Dependency Theory (25).

**Figure 1:**
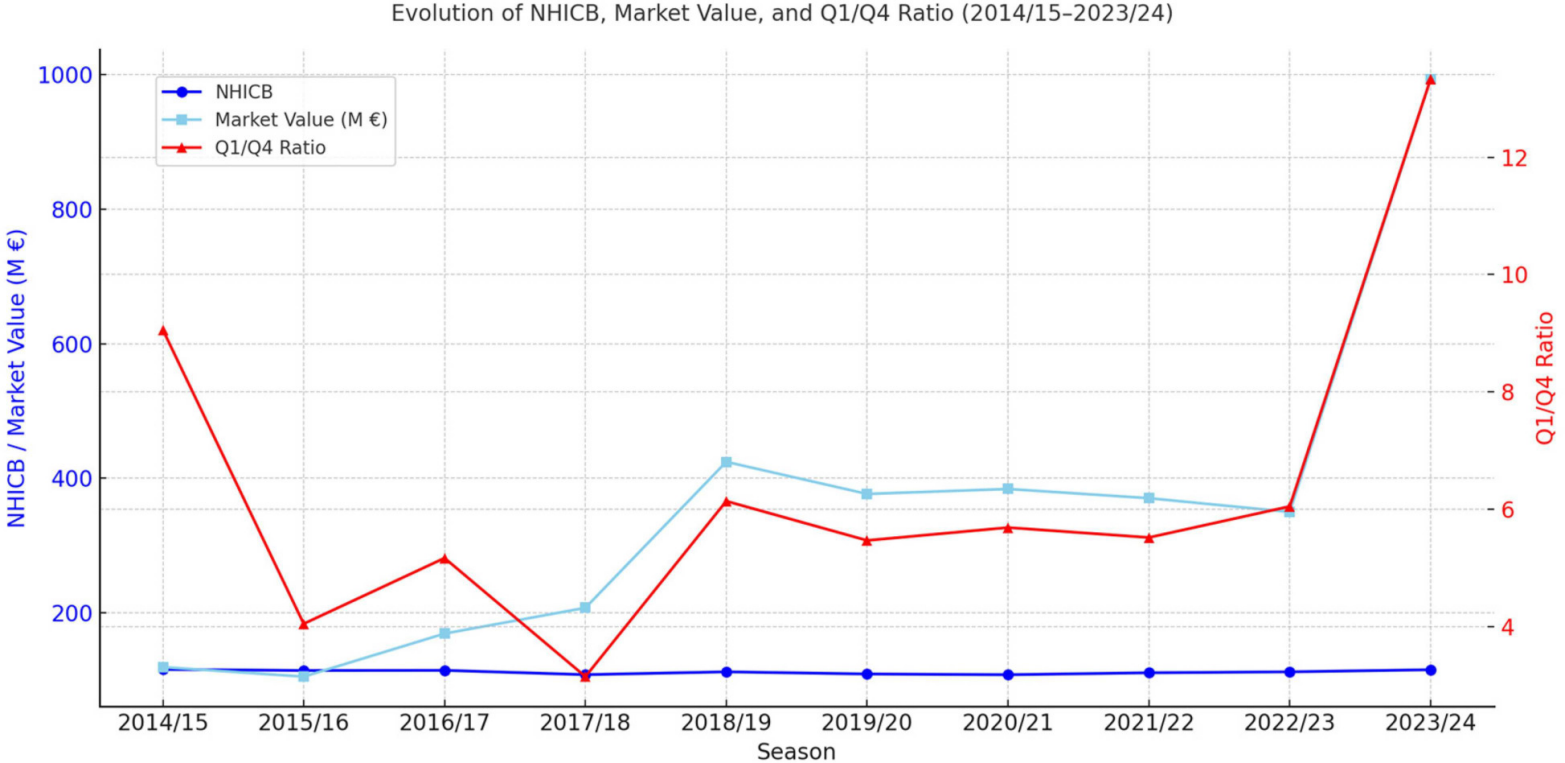
Evolution of NHICB, market value and Q1/Q4 SPL. Source: Authors’ elaboration.

From the 2018/19 season onwards, the SPL entered a second phase, marked by its initial expansion to 16 teams and a growing role of the PIF. This structural change paved the way for further transformation, culminating in the expansion to 18 teams in the 2023/24 season, along with a significant increase in public investment and international visibility. As shown in Table 3 and visualized in Figure 1, this period saw a sharp increase in market value—reaching nearly €1 billion by 2023/24—but also a considerable rise in financial inequality, with the Q1/Q4 ratio peaking at 13.33. The NHICB also increased to 115.3, reflecting a deterioration in competitive balance. Figure 1 clearly illustrates this divergence between economic growth and competitive equity, highlighting how public capital concentrated in a few elite clubs has contributed to structural inequality within the league.

At the same time, this period has coincided with an improvement in sporting performance at the international level. Saudi Arabian clubs have significantly enhanced their results in the AFC Champions League, with Al-Hilal winning the title in 2019 and 2021 (and being finalists in 2022), and Al-Ahli securing their first continental trophy in 2025. Clubs such as Al-Nassr have also consistently reached the latter stages of the competition. Notably, these three clubs are also the highest-valued teams in the league, as noted in the introduction section.

To test the relationship among the main variables, a Spearman correlation analysis was carried out (see Table 4). The results show a strong positive correlation between market value and the Q1/Q4 ratio (*ρ* = 0.721, *p* = 0.019), confirming that financial growth has coincided with increasing inequality. Moreover, a statistically significant correlation was also observed between NHICB and Q1/Q4 (*ρ* = 0.636, *p* = 0.048), suggesting that financial disparity among clubs negatively impacts competitive balance. In contrast, the correlation between NHICB and overall market value was weak and not statistically significant (*ρ* = 0.127, *p* = 0.731), suggesting that the league's financial expansion does not inherently improve or harm competitive balance—it is the distribution of resources, not their volume, that matters.

**Table 4:** Spearman's correlation analysis.

	NHICB	MARKET VALUE	Q1/Q4
NHICB	1	0.127 *(p* *=* 0.731)	0.636 (*p* = 0.048*)
MARKET VALUE		1	0.721 (*p* = 0.019*)
Q1/Q4 ratio			1

Source: Authors’ elaboration.

*Indicate statistically significant coefficient at 5% levels.

## 5 Discussion

The findings suggest that Saudi Vision 2030 has served as a powerful catalyst for football development in the Kingdom, particularly in terms of attracting international talent and enhancing global visibility. The SPL's international visibility has experienced a notable increase; according to Google Trends data, global search interest in the Saudi Pro League increased more than 30-fold between the opening months of the 2022–23 and 2023–24 seasons. In several markets, it even surpassed search volumes for some major European leagues such as Serie A, Ligue 1, and the Bundesliga (55). However, the benefits have not been evenly distributed. The current model may risk further polarizing the league between a small group of financially privileged clubs and the rest. Football is unique in that greater competition tends to drive higher audience interest and financial returns (56).

Because the Saudi Pro League is actively positioning itself as a future peer to Europe's “Big Five”, benchmarking its competitive balance against those leagues offers the most relevant yardstick for judging its progress and ambitions. The “Big Five” label comes from UEFA's annual country-coefficient ranking, which has consistently placed the English Premier League, Spanish LaLiga, German Bundesliga, Italian Serie A, and French Ligue 1 in the top five slots (57, 58). This coefficient, updated every season aggregates each nation's results in the Champions League, Europa League, and Conference League over the preceding five years; a federation's position then determines both the number of clubs it may enter and the round at which they start. Currently, the top four associations receive four direct places in the group stage of the UEFA Champions League. Against this benchmark, the average NHICB of the SPL is slightly higher than those reported for the European “Big Five” (Table 5). These leagues have also experienced a declining balance following the implementation of Financial Fair Play (50). In both Saudi and European contexts, this imbalance is more strongly associated with concentrated investment rather than liberalization or the arrival of foreign players (40, 59). European experience suggests that liberalization measures—like the Bosman ruling—can enhance balance by promoting player mobility and redistributing talent. By contrast, FFP-style regulations may entrench existing inequalities. As Peeters and Szymanski (35) note, FFP often consolidates the dominance of wealthy clubs, restricting financial flexibility for smaller ones and thus exacerbating disparities.

**Table 5:** NHICB average in the Big 5 leagues.

League	NHICB
Premier League (England)	111.35
LaLiga (Spain)	109.73
Bundesliga (Germany)	109.27
Calcio (Italia)	111.32
Ligue 1 (France)	107.99
Saudi Pro League	111.61

Source: Author's elaboration based on (50).

Thus, the results show that the implementation of the Saudi Vision 2030 programme has been a significant boost for football in Saudi Arabia in its bid to turn the SPL into a global benchmark. The SPL has led the AFC Club Competitions Ranking for the fourth consecutive season since 2021–22, achieving an estimated score of approximately 119.96 points in the 2024–25 edition, surpassing traditionally stronger Asian leagues such as those of Japan and South Korea^3^ (60) In this sense, the plan is working in attracting international talent and increasing the League's visibility, but there is a risk of increasing the League's polarisation between a few teams that have access to these international players and the rest. Football is one of the few industries in which competition incentivises the generation of windfall profits (56) as the greater the rivalry, the greater the public interest.

Between 2014/15 and 2017/18, the SPL experienced balanced and sustainable growth, where increasing market value was accompanied by a narrowing financial gap. From 2018/19, however, growth became more accelerated and uneven due to PIF investments. With four major clubs under its control, the PIF has driven competitive divergence. These investments raise concerns about long-term financial sustainability, particularly as they do not seem to be guided by market criteria. Although the Saudi government aims to liberalize the SPL, it is doing so under a guided model using the PIF as a launchpad to attract international talent and improve infrastructure. The full transition to a privatized system will depend on macroeconomic stability and investor confidence. As shown in the Iranian context, transitioning to mixed or private ownership can strengthen club governance and financial stability (33). However, without reform mechanisms that diversify investment sources and incentivize performance, the current model may prove unsustainable in the long term.

Domestically, the SPL's expansion has not yet translated into significant increases in match attendance (Table 6), suggesting limited engagement at the community level. This is consistent with studies on globalisation in football which argue that, while financial investments are important for the development of football, the cultural and social links that communities have with the sport are crucial for its long-term growth and anchoring (21). At the same time, football can amplify the visibility and agency of local communities by creating spaces for participation, inclusion, and contestation. Lysa (61) documents how women in Qatar have leveraged football fandom and related initiatives to negotiate public presence and challenge prevailing patriarchal norms. These insights imply that deepening grassroots engagement may be as important as headline investment if the SPL is to consolidate domestic support.

**Table 6:** Evolution of the number of spectators per match.

Season	2014/15	2016/17	2018/19	2020/21	2022/23	2023/24
Attendance	9,676	7,053	9,493	8,214	10,197	8,488

Source: Own elaboration based on data of transfermarkt.es.

At the international level, however, the PIF's sports strategy—combined with initiatives like LIV Golf—has significantly enhanced Saudi Arabia's global profile. Whether this represents a sincere effort to reposition the Kingdom or a case of “sportswashing” remains open to debate (20, 62). Either way, it highlights the strategic importance of football as a soft power tool under Vision 2030.

However, we have made it clear that there is no consensus on the meaning of the term sportswashing and research on its effects remains limited (63). Therefore, its uncritical use to discredit sporting events or countries is problematic, as it obscures more than it clarifies and is not always empirically supported (1, 2). The same is true of the use of sport as an instrument of soft power, as it is not neutral, given that it can legitimize political projects, but also expose them (13, 24). Faced with this, approaches such as that of Jarvie (64) suggest that the future should move towards using sport as a means of cultural relations and the construction of common goods, rather than as a propaganda showcase, which would seem to be more aligned with the pillars that make up SA's Vision 2030 (6).

On the scope of a theoretical framework, RDT may help explain Saudi clubs' structural dependence on state funding, but its link to other revenue streams such as broadcast rights, commercial agreements, and the growing participation of fans on social media, who, along with the government, form part of each club's interest groups, is difficult to explain. This has been shown to be better interpreted from the perspective of Stakeholder Theory (65).

The significant presence of public funds in the Saudi Arabian league clearly influences the league's sustainability if it were to be controlled by private capital or the market. However, this government control strengthens the maturing process to capture global attention, paving the way for future market opening, as was the case with clubs in European leagues, which were also sports associations heavily dependent on resources from the regional governments to which they belonged before becoming sports corporations or listing on the market. Indeed, clubs such as FC Barcelona, Real Madrid, Athletic Bilbao, and Osasuna still retain this legal status, and many others still depend wholly or partially on the sports facilities of their regional governments (66).

In this maturation horizon, RDT also presents limitations when explaining the conflicting interests that may arise between new actors involved in the league and the Saudi government, for which Agency Theory has proven to offer an appropriate framework (67–70).

Additionally, the Saudi league may be replicating the traditional management model that prioritizes success on the field over the financial sustainability of clubs, which is beyond the scope of the RDT, as well as justifying the failure of UEFA and some national league controls to eradicate this behavior (67, 68), to which the Soft Budget Constraints (SBC) theory could offer support, given the certainty of clubs being bailed out in the event of difficulties (71).

## 6 Conclusions and future research

Over the past decade, Saudi Arabia, along with other countries in the Arabian Peninsula such as Qatar and the UAE, has extended its influence in the global football arena through sponsorships and acquisitions of European football clubs, hosting major international sporting events or making significant investments in sports media to challenge the traditional dominance of international sports organizations. In the case of football, the expansion of the SPL has been hastened by the involvement of the Saudi Arabian Public Investment Fund (PIF), which has injected substantial funds into the four clubs under its control. This paper sought to analyze the impact of this SPL development strategy on the competitive balance, arriving at the following conclusions:

Competitive balance is closely tied to the presence of economic equilibrium among participating clubs, fostering an environment of equal opportunity. The expansion of the SPL, whether through the enlargement of the competition or the introduction of FIP investment, widens the economic disparity between wealthier and less affluent clubs.

Nevertheless, the repercussions of imbalances stemming from the inclusion of new clubs in the competition tend to be rectified as seasons progress, whereas those resulting from investments injected into select clubs persist over time, as long as these financial injections remain recurrent.

The passion for football in other countries of the world is not only determined by the quality of the spectacle, but also by cultural factors and people's identification to their city or neighbourhood club, which are not easily transferable to other geographical contexts.

The investment plan of the PIF does not seem to be based on the financial viability of the project. Conversely, Saudi Arabia's football development plan seems to be outward-focused, serving as a component of the Saudi Vision 2030 strategic framework aimed at economic diversification and promoting a more moderate image of the Kingdom. Leveraging the international impact of sports can help normalize perceptions of the country in other regions of the world, attracting foreign investment and fostering tourism development. It's possible that there's an element of “sporstwashing” in Saudi Vision 2030, using sports to divert attention from other human rights issues in the Kingdom, but as we've already made clear from the outset, the questionable propagandistic and un-academic nature of this matter is not the topic at hand. In any case, similar to the UN's 2030 Agenda, the mere act of acknowledging areas for improvement plants the seeds of change that will eventually bear fruit.

The RSD has provided a framework to go some way toward explaining the phenomenon of the Saudi League, which is heavily dependent on public funds. However, its scope is limited, so a more exhaustive analysis would necessarily require a multi-theoretical framework (72).

This paper contributes to the literature by providing a comparative assessment of competitive balance in the SPL vs. the five major European leagues using the NHICB. It also links state investment under Vision 2030 and PIF funding to league outcomes and situates the SPL within the broader Gulf political context, where sport serves diversification and nation-branding goals. Together, these elements integrate performance metrics with institutional and political-economy perspectives.

Further research is needed to explore the financial management of SPL clubs. A more detailed analysis of their financial performance, particularly in comparison to traditional European football clubs, would provide a deeper understanding of the sustainability of the SPL's expansion. Additionally, research should examine the revenue structures and governing models within the SPL, focusing on the impact of PIF investment on financial outcomes, and the potential long-term implications for competitive balance.

Finally, future studies could assess the broader implications of Saudi Arabia's sports investment on regional sports development, the global football landscape, and its alignment with the nation's socio-political objectives. This would enhance the understanding of how sports investments can be leveraged as a tool for economic diversification and international diplomacy.

## Data Availability

The raw data supporting the conclusions of this article will be made available by the authors, without undue reservation.

## Footnotes

^1^Transfermarkt (https://www.transfermarkt.es/saudi-pro-league/startseite/wettbewerb/SA1/plus/?saison_id=2023, last accessed 29 July 2025).

^2^Market value data for each club were sourced from the specialized platform Transfermarkt (http://www.transfermarkt.es), as consulted for each relevant season.

^3^See https://www.the-afc.com/en/more/afc_ranking/mens_ranking.html
